# Immune response to co-administration of measles, mumps, and rubella (MMR), and yellow fever vaccines: a randomized non-inferiority trial among one-year-old children in Argentina

**DOI:** 10.1186/s12879-023-08114-1

**Published:** 2023-03-17

**Authors:** Carla Vizzotti, Jennifer B. Harris, Analía Aquino, Carolina Rancaño, Cristian Biscayart, Romina Bonaventura, Andrea Pontoriero, Elsa Baumeister, Maria Cecilia Freire, Mirta Magariños, Blanca Duarte, Gavin Grant, Susan Reef, Janeen Laven, Kathleen A. Wannemuehler, Alba Maria Ropero Alvarez, J. Erin Staples

**Affiliations:** 1 Dirección de Control de Enfermedades Inmunoprevenibles, Ministerio de Salud de Argentina, Buenos Aires, Argentina; 2 Global Immunization Division, Centers for Disease Control and Prevention (CDC), Atlanta, GA USA; 3 Departamento de Virología, Instituto Nacional de Enfermedades Infecciosas, Administración Nacional de Laboratorios e Institutos de Salud “Dr. Carlos Malbrán” (ANLIS), Buenos Aires, Argentina; 4 Pan American Health Organization (PAHO), Buenos Aires, Argentina; 5 Programa Provincial Regular de Inmunizaciones, Ministerio de Salud de Misiones, Posadas, Argentina; 6 Division of Vector-Borne Diseases, Centers for Disease Control and Prevention (CDC), Fort Collins, CO USA; 7 Biostatistics and Medical Informatics, University of Wisconsin, Madison, WI USA; 8 Pan American Health Organization (PAHO), Washington, DC USA

**Keywords:** Measles, Mumps, Rubella, Yellow fever, Vaccine, MMR

## Abstract

**Background:**

In yellow fever (YF) endemic areas, measles, mumps, and rubella (MMR), and YF vaccines are often co-administered in childhood vaccination schedules. Because these are live vaccines, we assessed potential immune interference that could result from co-administration.

**Methods:**

We conducted an open-label, randomized non-inferiority trial among healthy 1-year-olds in Misiones Province, Argentina. Children were randomized to one of three groups (1:1:1): Co-administration of MMR and YF vaccines (MMR_1_YF_1_), MMR followed by YF vaccine four weeks later (MMR_1_YF_2_), or YF followed by MMR vaccine four weeks later (YF_1_MMR_2_). Blood samples obtained pre-vaccination and 28 days post-vaccination were tested for immunoglobulin G antibodies against measles, mumps, and rubella, and for YF virus-specific neutralizing antibodies. Non-inferiority in seroconversion was assessed using a -5% non-inferiority margin. Antibody concentrations were compared with Kruskal-Wallis tests.

**Results:**

Of 851 randomized children, 738 were correctly vaccinated, had ≥ 1 follow-up sample, and were included in the intention-to-treat population. Non-inferior seroconversion was observed for all antigens (measles seroconversion: 97.9% in the MMR_1_YF_1_ group versus 96.3% in the MMR_1_YF_2_ group, a difference of 1.6% [90% CI -1.5, 4.7]; rubella: 97.9% MMR_1_YF_1_ versus 94.7% MMR_1_YF_2_, a difference of 3.3% [-0.1, 6.7]; mumps: 96.7% MMR_1_YF_1_ versus 97.9% MMR_1_YF_2_, a difference of -1.3% [-4.1, 1.5]; and YF: 96.3% MMR_1_YF_1_ versus 97.5% YF_1_MMR_2_, a difference of -1.2% [-4.2, 1.7]). Rubella antibody concentrations and YF titers were significantly lower following co-administration; measles and mumps concentrations were not impacted.

**Conclusion:**

Effective seroconversion was achieved and was not impacted by the co-administration, although antibody levels for two antigens were lower. The impact of lower antibody levels needs to be weighed against missed opportunities for vaccination to determine optimal timing for MMR and YF vaccine administration.

**Trial Registration:**

The study was retrospectively registered in ClinicalTrials.gov (NCT03368495) on 11/12/2017.

**Supplementary Information:**

The online version contains supplementary material available at 10.1186/s12879-023-08114-1.

## Background

Thirteen countries in the Americas and 34 in Africa are categorized as high risk for yellow fever (YF), either nationwide or within subnational areas. Almost all YF endemic countries administer both YF vaccine and measles-containing vaccine (MCV), i.e., measles, mumps, and rubella (MMR), measles-rubella (MR), or measles vaccine, via their national immunization programs. Because these are all live vaccines, the World Health Organization (WHO) recommends that they are administered at the same time (co-administered) or individually at least four weeks apart to prevent potential immune interference [1, 2]. From a programmatic perspective, coadministration provides protection for all four diseases at the earliest possible age, maximizes the efficient use of healthcare resources, and prevents children from potentially missing a vaccine dose should they not return for a later vaccination visit [3].

Previous studies did not show an impact on reactogenicity and immunogenicity when measles and YF vaccines were co-administered [4]. However, a study from Brazil published in 2011 found that immunogenicity against mumps, rubella, and YF is compromised when MMR and YF vaccines are co-administered [5]. Based on these findings, the WHO, with the approval of the Strategic Advisory Group of Experts (SAGE) on Immunization, recommended additional studies be conducted to evaluate the co-administration of these vaccines [1, 2].

Since 2007, the Argentinean Ministry of Health (MOH) has recommended that YF vaccine be administered in part or all of the three northeastern provinces that are considered endemic for YF [6]. Initially, YF vaccine was given together with MMR at 12 months of age, but the schedule was changed in 2014 when the National Commission for Immunization shifted YF vaccine to 18 months of age because of concerns over potential adherence issues with increasing numbers of injectable vaccines at the same visit [7]. In the years following this change, YF vaccine coverage declined to levels 15–40% lower than MMR coverage [8]. Currently, other countries in the Americas either co-administer the vaccines at 12 months of age or provide MMR at 12 months with YF vaccine at 9, 15, or 18 months [9]. In Africa, 22 countries co-administer YF vaccine with MCV at 9 months of age [7]. Finally, YF and MMR vaccines are often co-administered to children in non-endemic areas when they travel to YF endemic areas.

From 2015 to 2018, the Argentinean MOH, Pan-American Health Organization (PAHO), and the U.S. Centers for Disease Control and Prevention (CDC) conducted a randomized controlled trial to evaluate the immune response to MMR and YF vaccines when co-administered to children aged 12–13 months. The study’s primary objective was to assess whether seroconversion against measles, rubella, mumps, and YF antigens following co-administration of MMR and YF vaccines was non-inferior to seroconversion when the vaccines were administered as the first vaccine in a series separated by four weeks (individual administration). Secondary objectives were to assess: (a) the magnitude of antibody responses to each antigen when the vaccines were co-administered compared with individual administration; (b) seroconversion and the magnitude of antibody responses when the vaccines were co-administered compared with administration as the second vaccine in a series separated by four weeks; and (c) seroconversion and the magnitude of antibody responses when the vaccines were administered as the second vaccine compared with administration as the first vaccine in a series separated by four weeks.

## Methods

### Study design

This phase IV, non-blinded, randomized non-inferiority trial took place at four urban health centers and three satellite clinics in the cities of Posadas, Eldorado, and Oberá in Misiones Province, Argentina. Centers were selected based on location in a YF endemic area, accessibility, capacity to carry out the protocol, and average number of children vaccinated each month. The study protocol was approved by the medical ethics committees of the MOH of Argentina and PAHO. In accordance with human subjects’ review procedures at the CDC, it was determined that the CDC was not formally engaged in this human-subjects research. The study was retrospectively registered in ClinicalTrials.gov (NCT03368495) on 11/12/2017 and the national regulatory agency for medical technology (ANMAT) was informed.

### Participants

Caregivers of children were informed about the study when they presented for their 10, 11, and 12-month check-ups. Those who indicated interest were scheduled for their child’s 12-month vaccinations on one of the twice-weekly study days. Eligible children were aged 12–13 months, clinically healthy, and up to date on prior immunizations. Children with a prior history of MMR or YF vaccination, contraindications to MMR or YF vaccines, or a history of having measles, mumps, rubella, or YF disease were excluded. A parent or legal guardian provided written informed consent for their child’s participation.

### Randomization and masking

Children were randomly assigned (1:1:1) to one of three groups: Co-administration of MMR and YF vaccines (MMR_1_YF_1_), MMR followed by YF vaccine four weeks later (MMR_1_YF_2_), or YF vaccine followed by MMR four weeks later (YF_1_MMR_2_). Investigators who had no participant contact generated the randomization sequence in blocks of six, stratified by health center, using R software (Version 3.2.2). Assignments were placed in sealed, numbered, opaque envelopes provided to each study site. Study clinic staff assigned envelopes to children in the order that they enrolled at the site. Neither study staff nor parents were blinded to the group assignment.

### Procedures

At the baseline visit, study physicians performed a brief clinical history and exam. A 5 ml venous blood sample was collected, and children were vaccinated with MMR, YF vaccine, or both depending on their randomization group. Vaccination procedures followed the 2012 edition of the Argentinian National Immunization Guidelines [10]. Vaccines used during the study were those provided through the immunization program and were procured using PAHO´s Revolving Fund. Two MMR vaccines were used throughout the study: one manufactured by Sanofi Pasteur (Schwarz strain for measles; Urabe AM-9 strain for mumps; Wistar RA 27/3 M strain for rubella) and one manufactured by Merck (Enders’ attenuated Edmonston strain for measles; Jeryl Lynn strain for mumps; Wistar RA 27/3 strain for rubella). Two YF vaccines were used: 17D-204 (Sanofi Pasteur) and 17DD (Bio-Manguinhos). Study subjects received 0.5 ml of MMR vaccine administered subcutaneously in the right arm and 0.5 ml of YF vaccine injected subcutaneously in the left arm.

At 28–35 days (Visit 2), all children returned for a follow-up venous blood draw of up to 5 ml. Children in the MMR_1_YF_2_ and YF_1_MMR_2_ vaccination groups also received the individual vaccine they had not received at Visit 1. Children in the MMR_1_YF_1_ group did not receive additional vaccines. A third visit (Visit 3) was conducted 28–35 days after Visit 2 for children in the MMR_1_YF_2_ and YF_1_MMR_2_ groups to have their final venous blood draw.

Samples were refrigerated at the study sites and transported within 24 h to the Central Blood Bank of Misiones Province. Samples were centrifuged at either the study site or the Blood Bank, and serum was separated and stored at 2–8 °C at the Blood Bank. Serum was flown to the Instituto Nacional de Enfermedades Infecciosas, Administración Nacional de Laboratorios e Institutos de Salud “Dr. Carlos Malbrán” (ANLIS) in Buenos Aires twice a week, where serum was aliquoted into separate cryovials for MMR and YF testing and then stored at -70 °C until testing was performed.

Parents were informed about potential local and systemic reactions to vaccination and asked to notify the study staff of any serious adverse event, as defined by national authorities [10]. At Visits 2 and 3, parents were asked about local reactions and illnesses during the interval since the prior visit.

Measles and rubella testing was performed at the Servicio de Virosis Respiratorias and mumps testing was performed at the Servicio de Neurovirosis, both within the Instituto Nacional de Enfermedades Infecciosas, ANLIS. Quantitative enzyme immunoassay (ELISA) IgG kits manufactured by Siemens (Marburg, Germany) were used to measure IgG antibody concentrations against measles, mumps, and rubella.

YF testing was performed at the CDC Arbovirus Diseases Laboratory, Fort Collins, Colorado, USA. Paired baseline and follow-up samples were tested for the presence of neutralizing antibodies against YF 17-D 204 virus, using the plaque reduction neutralization test with cut-offs of 50% (PRNT_50_) and 90% (PRNT_90_), as previously described [11]. To ensure consistency of the PRNT results, testing was performed at the end of the study over several weeks using a standard internal control. PRNT_50_ titers are presented in the main analyses because this cutoff is typically used in flavivirus vaccine trials and is recommended by WHO for establishing sufficient virus-neutralizing antibodies in immunogenicity studies conducted by vaccine manufacturers [12].

### Outcomes

The primary outcome assessed was seroconversion (being seronegative at baseline and seropositive post-vaccination) to the antigens in the vaccines received at the baseline visit. A secondary outcome assessed antibody levels post-vaccination, defined as IgG antibody concentrations for measles, mumps, and rubella and neutralizing antibody titers for YF, to the antigens in the vaccines received at the baseline visit. Additional secondary outcomes included seroconversion and antibody levels following MMR and YF vaccines received at Visit 2. The cut-offs used for seropositivity were as follows: measles concentrations > 150 mIU/ml; rubella concentrations > 4 IU/ml; mumps concentrations ≥ 231 U/ml; and YF PRNT_50_ titers ≥ 10. Baseline samples were tested for antibodies to all antigens. Follow-up samples were tested only for the antigens in the vaccine received at the previous visit; hence, follow-up testing was conducted 28–35 days post-vaccination for all vaccines received, regardless of the randomization group.

Adverse event monitoring followed national guidelines and classification criteria described elsewhere [10]. Serious adverse events were those that resulted in hospitalization or death.

### Statistical analysis

For sample size calculations, we estimated that at least 95% of the children receiving the vaccines sequentially would seroconvert to measles, rubella, and yellow fever [1, 2, 13]. Based on measles subject matter experts’ recommendations, we selected a non-inferiority margin of -5% because the high infectivity of measles requires high levels of seroconversion to support existing elimination goals. For consistency, the same margin for rubella and YF was used. Power was set at 0.80 and the significance level at 0.05 (one-tailed). This resulted in a sample size of 234 per group. Accounting for 20% attrition, the final sample size was 294 per group and 882 in total. Sample size calculations were not powered to show non-inferiority against mumps, given that seroconversion against mumps is not consistently at or above 95% [14].

We assessed if seroconversion following co-administration was non-inferior to seroconversion following the administration of individual vaccines as either the first or second vaccine in a series separated by four weeks. We also assessed whether seroconversion following administration as the second vaccine in the series was non-inferior to seroconversion following administration as the first vaccine. Non-inferiority was assessed by comparing the lower bound of the 90% Farrington-Manning confidence interval (CI) for the difference in seroconversion to the non-inferiority margin of -5%. We assessed the pairwise differences in the distribution of antibody concentrations/titers with Wilcoxon rank-sum tests, using a significance level of 0.05. Since all hypotheses were described *a priori*, we did not make any adjustments to account for testing multiple hypotheses. We used violin plots to visualize the distribution of antibody titers and reverse cumulative distribution curves to visualize the proportion of children with at least a given level of antibody/response. We then compared the type and frequency of safety events among the three groups.

Analyses are presented for two populations: The *intention-to-treat (ITT) population* included all children that were correctly vaccinated, had an adequate baseline sample, and had at least one follow-up sample. The *per-protocol (PP) population* was further limited to include only samples from the follow-up visits completed within the specified time frame of 28–35 days following the prior vaccination. We excluded children from antigen-specific analyses if they were seropositive to that antigen at baseline. Data were analyzed using SAS (version 9.4) and R (version 3.5.1).

## Results

**Fig. 1:**
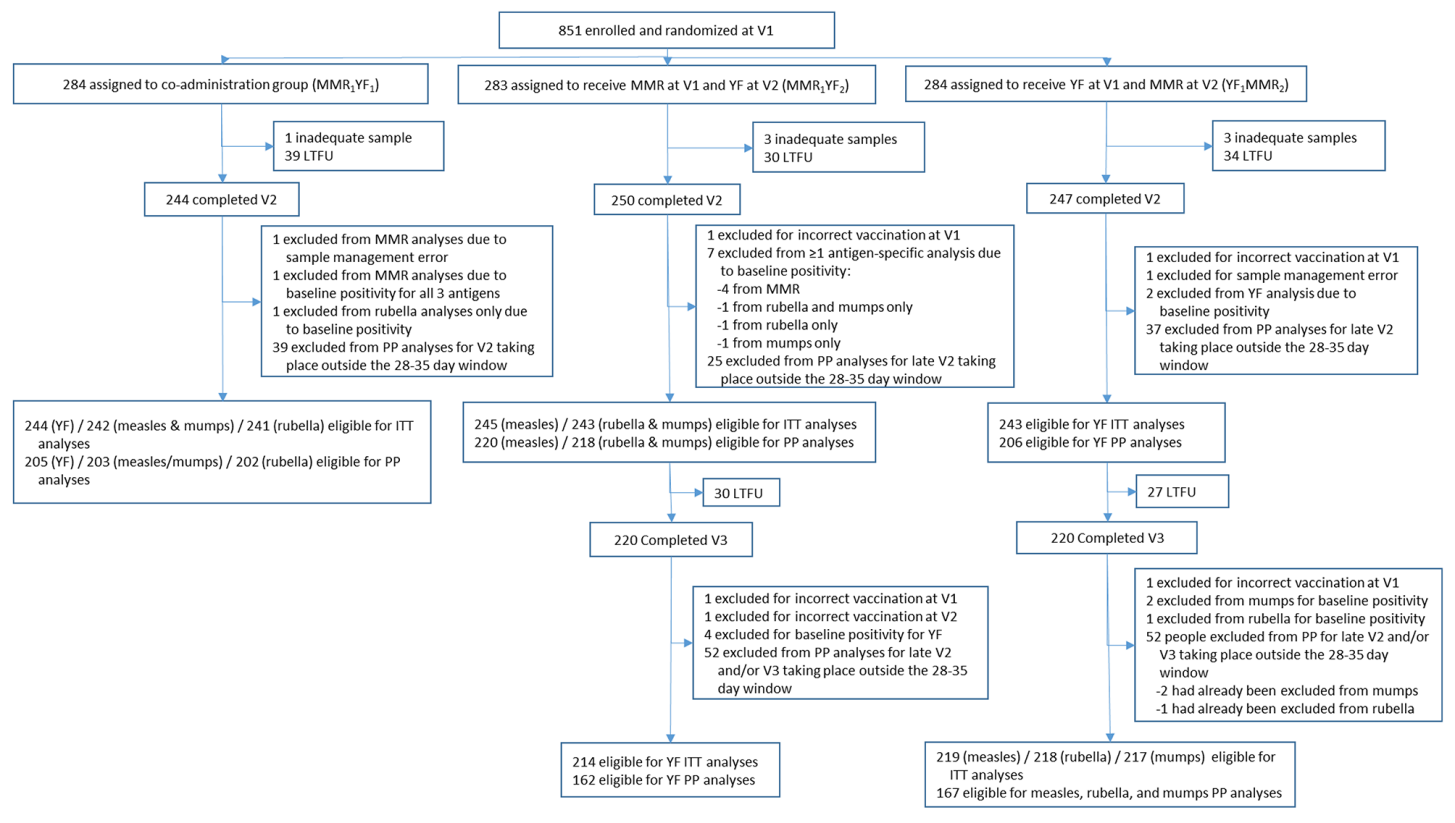
Trial Profile. Legend: V1 = Visit (1) V2 = Visit (2) V3 = Visit (3) MMR = Measles, mumps, and rubella vaccine. YF = Yellow fever vaccine. LTFU = Lost to follow-up. ITT = Intention-to-treat. PP = Per protocol

From November 23, 2015 to April 16, 2018, 851 children were enrolled and randomized (Fig. 1); the number of children screened was not systematically collected. A total of 741 (87.0%) children completed the second visit, and 738 (86.7%) were included in the ITT population. Three children were excluded due to incorrect vaccination at the baseline visit (n = 2) and a sample management error (n = 1). In addition, children were excluded from antigen-specific analyses if they were seropositive at baseline (n = 4 for measles; n = 8 for rubella; n = 7 for mumps; n = 2 for YF). An additional sample management error resulted in the exclusion of one child’s data from the measles, mumps, and rubella analyses. An additional 101 children were excluded from the PP analyses because their second visit took place outside of the 28–35-day window: 15 children with visits before 28 days and 86 children with visits after 35 days. A total of 497 children required a third visit (MMR_1_YF_2_ and YF_1_MMR_2_); 440 (88.5%) of these children completed Visit 3. Of the 440 children, 433 (87.1%) had results included in ITT analyses for at least one antigen after exclusion for vaccination errors (n = 3) and baseline positivity for antigens received in their visit 2 vaccination (n = 1 for rubella; n = 2 for mumps; n = 4 for YF). An additional 69 children were excluded from per-protocol analyses involving samples collected at Visit 3 because their visit took place outside of the 28–35-day window: 14 children with visits before 28 days and 55 children with visits after 35 days. Per protocol analyses with Visit 3 samples included only those participants who attended both follow-up visits within the 28–35-day window; hence, a total of 104 children were excluded for a late visit 2 or 3 (Fig. 1 and Additional Fig. 1).

The baseline demographics of the ITT population are shown in Table 1. Slightly more than half (52.7%) of the study participants were male, mean weight was 9.7 kg, and 79.8% were still breastfeeding, though not exclusively.

**Table 1:** Baseline Characteristics of Participants Completing at Least One Follow-Up Visit

	*MMR*_*1*_*YF*_*1*_: MMR and YF at first visit (n = 244)	*MMR*_*1*_*YF*_*2*_: MMR at first visit and YF at second visit (n = 249)	*YF*_*1*_*MMR*_*2*_: YF at first visit and MMR at second visit (n = 245)	Overall (n = 738)
Mean Age, months (SD)	12.6 (0.3)	12.6 (0.3)	12.6 (0.3)	12.6 (0.3)
Mean Weight, kg (SD)	9.8 (1.3)	9.5 (1.2)	9.6 (1.0)	9.7 (1.2)
Mean Height, cm (SD)	74.7 (3.3)	74.1 (3.2)	74.4 (2.9)	74.4 (3.1)
	**n (%)**	**n (%)**	**n (%)**	**n (%)**
Male sex	117 (48.0)	134 (53.8)	138 (56.3)	389 (52.7)
Breastfeeding				
No	42 (17.2)	53 (21.3)	44 (18.0)	139 (18.9)
Yes, exclusively	4 (1.6)	6 (2.4)	0	10 (1.4)
Yes, not exclusively	198 (81.2)	190 (76.3)	201 (82.0)	589 (79.8)
Baseline seropositivity				
Measles	1 (0.4)	4 (1.6)	0	5 (0.7)
Rubella	2 (0.8)	6 (2.4)	2 (0.8)	10 (1.4)
Mumps	1 (0.4)	6 (2.4)	4 (1.6)	11 (1.5)
Yellow Fever (PRNT_50_)	0	5 (2.0)^†^	2 (0.8)	7 (1.0)

Data shown are n (%) or mean (standard deviation)

MMR = Measles, Mumps, and Rubella vaccine. YF = Yellow Fever vaccine. PRNT_50_ = Plaque Reduction Neutralization Test with 50% cut-off. kg = kilograms. cm = centimeters.

^†^ Only 4 (1.6%) positive at baseline using PRNT with 90% cut-off (PRNT_90_)

**Fig. 2:**
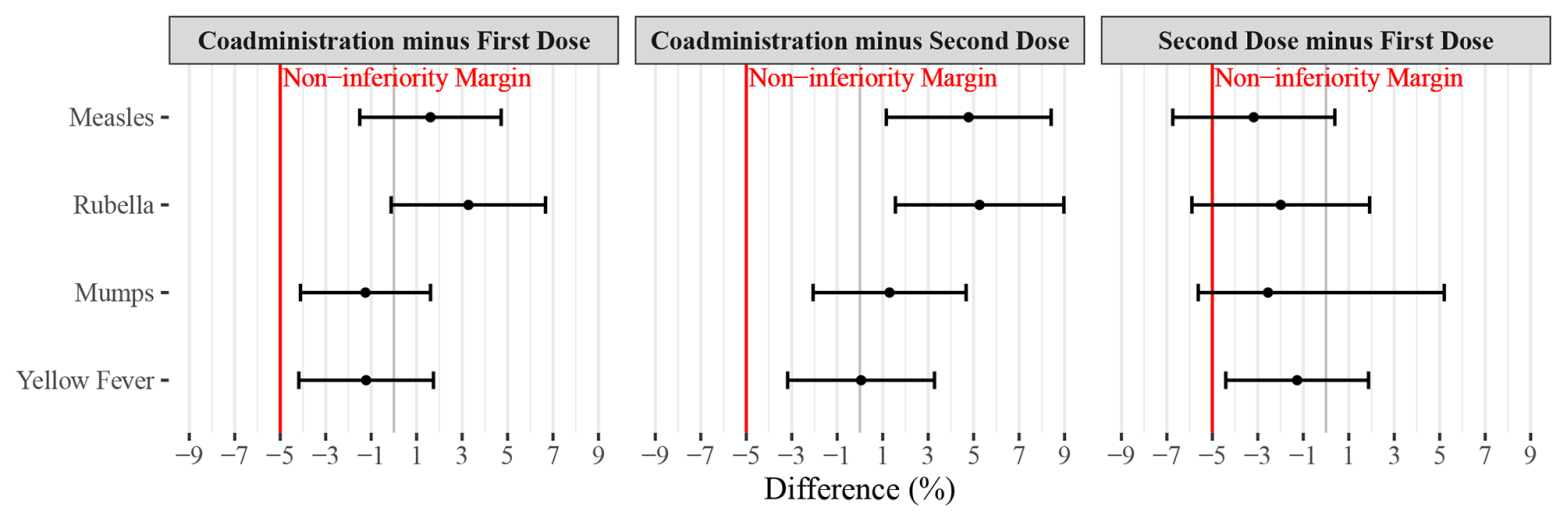
Differences in seroconversion between vaccination groups. The differences in seroconversion between vaccination groups are presented with Farrington-Manning 90% Confidence Interval around the estimated difference. The red line is the non-inferiority margin, set at -5%. “Coadministration minus First Dose” is the difference in the seroconversion following co-administration (MMR_1_YF_1_) minus the seroconversion following individual administration of the vaccine as the first vaccine in a 2-dose series (MMR_1_YF_2_ or YF_1_MMR_2_ ). “Coadministration minus Second Dose” is the difference in seroconversion following co-administration minus the seroconversion following individual administration as the second vaccine in a 2-dose series separated by four weeks. “Second Dose minus First Dose” is the difference in the seroconversion following individual administration as the second dose in a 2-dose series separated by four weeks minus the seroconversion following administration as the first vaccine in the series. In this intention-to-treat analysis, seroconversion following co-administration was non-inferior to seroconversion following individual administration as the first or second dose for all antigens. Non-inferiority in seroconversion was not shown for measles, mumps, and rubella when MMR was administered as the second vaccine as compared to when it was administered as the first vaccine in the series separated by four weeks

The primary objective assessed non-inferiority of seroconversion following co-administration compared with seroconversion following individual administration as the first vaccine in a series separated by four weeks. In the ITT population, seroconversion following co-administration was non-inferior for all antigens. Measles seroconversion was 97.9% in the co-administration group and 96.3% in the individual group (a difference of 1.6% [90% confidence interval (CI) -1.5, 4.7]). For the other antigens, seroconversion rates in the co-administration and individual groups, respectively, were 97.9% and 94.7% for rubella (a difference of 3.3% [-0.1, 6.7]); 96.7% and 97.9% for mumps (a difference of -1.3% [-4.1, 1.6]); and 96.3% and 97.5% for YF (a difference of -1.2% [-4.2, 1.7]). Similar rates of seroconversion were observed in the PP population; however, non-inferiority was not shown for YF with seroconversions of 96.1% in the co-administration group and 98.1% in the individual group (a difference of -2.0% [-5.1, 1.2]). Full results are shown in Table 2; Fig. 2.

**Table 2:** Seroconversion and post-vaccination antibody levels when YF and MMR are co-administered or administered individually

	MMR & YF vaccines co-administered*	MMR or YF vaccine administered individually as first vaccine in a series*	Difference in seroconversion or p-value for comparison of antibody titers/ concentrations†	Interpretation
**Measles**	*MMR*_*1*_ *YF*_*1*_	*MMR*_*1*_ *YF*_*2*_		
*Intention-to-treat analysis*	*(n = 242)*	*(n = 245)*		
Seroconversion	97.9 (95.3–99.1)	96.3 (93.2–98.1)	1.6 (-1.5–4.7)	Co-administration is non-inferior to individual administration
Antibody concentrations, mIU	2024 (1705–2402)	1638 (1323–2028)	0.175	No significant difference
*Per protocol analysis*	*(n = 203)*	*(n = 220)*		
Seroconversion	98.0 (95.0–99.2)	96.4 (93.0–98.2)	1.7 (-1.7–5.0)	Co-administration is non-inferior to individual administration
Antibody concentrations, mIU	1956 (1629–2348)	1569 (1253–1964)	0.188	No significant difference
**Rubella**	*MMR*_*1*_ *YF*_*1*_	*MMR*_*1*_ *YF*_*2*_		
*Intention-to-treat analysis*	*(n = 241)*	*(n = 243)*		
Seroconversion	97.9 (95.2–99.1)	94.7 (91.0–96.9)	3.3 (-0.1–6.7)	Co-administration is non-inferior to individual administration
Antibody concentrations, IU	35.8 (31.5–40.7)	40.8 (35.0–47.5)	0.006	Individual administration has higher concentrations
*Per protocol analysis*	*(n = 202)*	*(n = 218)*		
Seroconversion	97.5 (94.3–98.9)	94.5 (90.6–96.8)	3.0 (-0.7–6.8)	Co-administration is non-inferior to individual administration
Antibody concentrations, IU	32.2 (28.0–37.1)	39.4 (33.5–46.3)	0.001	Individual administration has higher concentrations
**Mumps**	*MMR*_*1*_ *YF*_*1*_	*MMR*_*1*_ *YF*_*2*_		
*Intent-to-treat analysis*	*(n = 242)*	*(n = 243)*		
Seroconversion	96.7 (93.6–98.3)	97.9 (95.3–99.1)	-1.3 (-4.1–1.6)	Co-administration is non-inferior to individual administration
Antibody concentrations, U	1807 (1470–2220)	2252 (1877–2701)	0.082	No significant difference
*Per protocol analysis*	*(n = 203)*	*(n = 218)*		
Seroconversion	96.6 (93.1–98.3)	98.2 (95.4–99.3)	-1.6 (-4.7–1.5)	Co-administration is non-inferior to individual administration
Antibody concentrations, U	1746 (1390–2192)	2319 (1926–2792)	0.037	Individual administration has higher concentrations
**Yellow Fever**	*MMR*_*1*_ *YF*_*1*_	*YF*_*1*_ *MMR*_*2*_		
*Intention-to-treat analysis*	*(n = 244)*	*(n = 243)*		
Seroconversion	96.3 (93.1–98.1)	97.5 (94.7–98.9)	-1.2 (-4.2–1.7)	Co-administration is non-inferior to individual administration
Antibody titers	219 (181–265)	340 (283–408)	< 0.0001	Individual administration has higher titers
*Per protocol analysis*	*(n = 205)*	*(n = 206)*		
Seroconversion	96.1 (92.5–98.0)	98.1 (95.1–99.2)	-2.0 (-5.1–1.2)	Non-inferiority of co-administration not shown
Antibody titers	225 (181–279)	373 (308–452)	< 0.0001	Individual administration has higher titers

*Data shown for seroconversion are percentages and 95% CI. Data shown for antibody concentrations are geometric mean concentrations and 95% CI. Data shown for antibody titers are geometric mean titer and 95% CI.

†Data shown are the difference in seroconversion and Farrington-Manning 90% CI or the p-value for Wilcoxon Rank Sum test comparing antibody titers/concentrations.

**Fig. 3:**
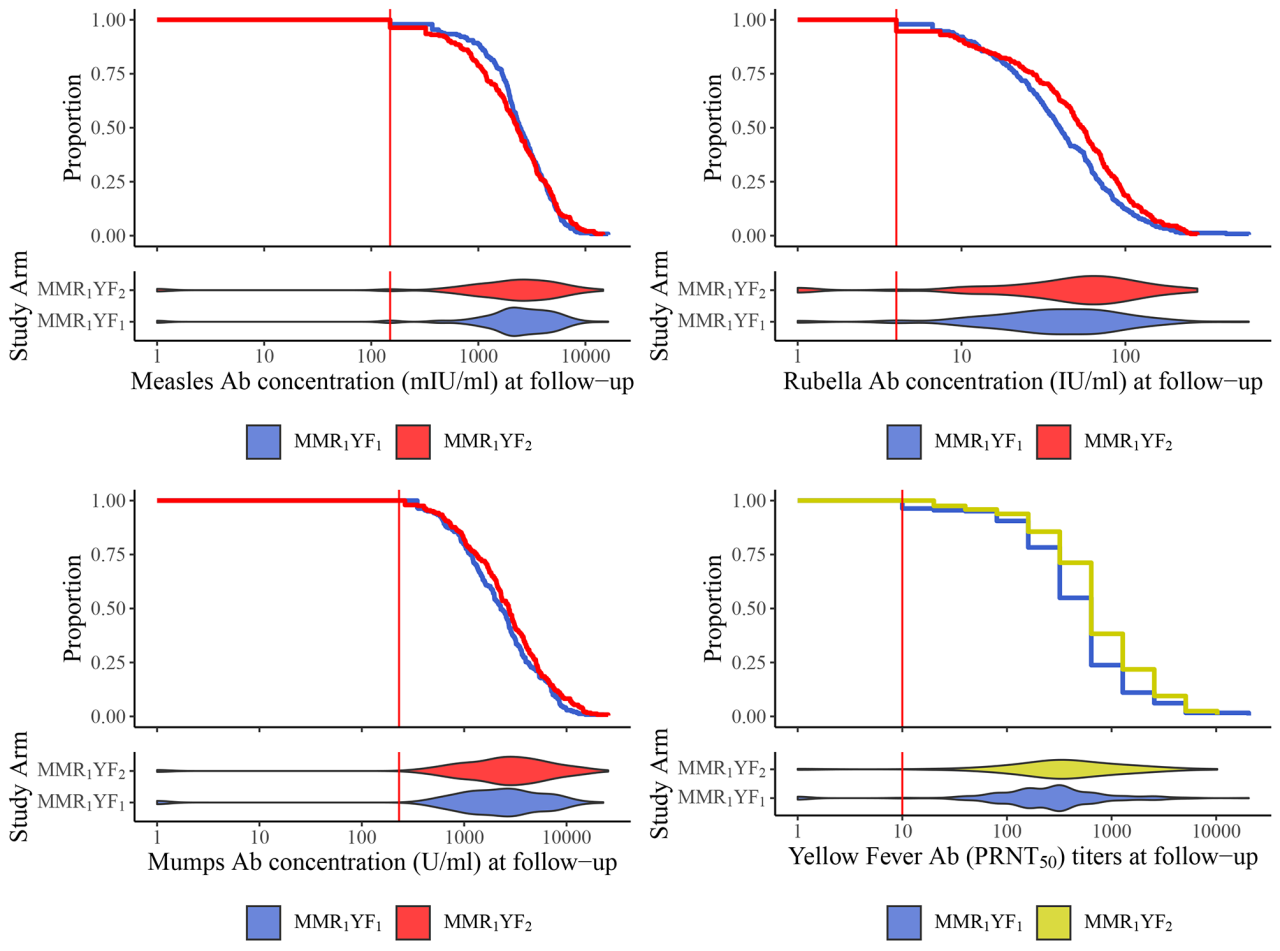
Measles, mumps, rubella, and yellow fever antibody concentrations/tiers when individually and co-administered. Reverse cumulative distribution and violin plots of measles, mumps, and rubella antibody (Ab) concentrations, and yellow fever Ab titers when vaccines are co-administered or administered as the first vaccine in a 2-vaccine series in the intention-to-treat population. The y-axis of the reverse cumulative distribution plots shows the proportion of participants with antibody concentrations/titers at least as high as the value shown in the x-axis. (Abbreviations: Ab = Antibody, U = Units, IU = International Units, PRNT = Plaque Reduction Neutralization Test)

A secondary objective was identical to the primary objective, except comparing antibody levels instead of seroconversion. In the ITT population, there was no significant difference in the measles antibody concentrations between the coadministration (geometric mean concentration [GMC] 2,024 mIU [95% CI 1,705, 2,402]) and the individual administration (1,638 mIU [1,323, 2,028]) groups (p = 0.175), nor in mumps concentrations between the coadministration (1,807 U [1,470, 2,220]) and the individual administration (2,252 U [1,877, 2,701]) groups (p = 0.082). However, there were differences in the magnitude of rubella and YF antibody responses. Rubella antibody concentrations were significantly lower following co-administration (35.8 IU [31.5, 40.7]) compared with individual administration (40.8 IU [35.0, 47.5]) (p = 0.006). YF neutralizing antibody titers were significantly lower following co-administration (219 [181, 265]) compared with individual administration (340 [283, 408]) (p < 0.0001). Similar results were observed in the PP population, except that mumps concentrations were also significantly lower following co-administration (1,746 U [1,390, 2,192]) compared with individual administration (2,319 U [1,926, 2,792]) (p = 0.037). Full results are shown in Table 2; Fig. 3.

The next secondary objective assessed non-inferiority of seroconversion following co-administration, compared to seroconversion following administration as the second vaccine. In the ITT population, co-administration led to higher rates of seroconversion for measles (a difference of 4.8% [90% CI 1.2, 8.4]) and rubella (a difference of 5.3% [1.2, 9.2]). For mumps and YF, seroconversion rates following co-administration were non-inferior to the rates achieved when administered as the second vaccine in the series (Fig. 2, Additional Table 1). When antibody concentrations were compared for these groups, there were significantly higher antibody concentrations (p = 0.018) obtained with co-administration for measles. However, significantly higher antibody levels were obtained for the other three antigens (mumps, rubella, and YF) when either the MMR or YF vaccine was administered second in the series rather than co-administered. All conclusions from the PP analyses aligned with the ITT analyses (Additional Table 1).

Finally, non-inferiority of seroconversion following administration of the second vaccine in a series separated by four weeks was assessed compared to seroconversion following administration of the first vaccine in the series. In the ITT population, non-inferiority was demonstrated only for YF (a difference of -1.3% [90% CI -4.4, 1.9]); however, non-inferiority was not demonstrated in the PP population for YF (Fig. 2, Additional Table 2). Non-inferiority was not demonstrated for seroconversion rates for measles, mumps, and rubella. However, there were no significant differences in antibody levels for these antigens or YF when the vaccine was second in the sequential series compared to when it was administered first in the series; this was consistent in ITT and PP populations (Additional Table 2).

Only seven adverse events were reported; none were serious. There was no significant difference in the number of adverse events among the study groups (2 in MMR_1_YF_1_ group; 3 in MMR_1_YF_2_ group; 2 in YF_1_MMR_2_ group).

## Discussion

In this study, we assessed seroconversion rates against measles, mumps, rubella, and YF antigens following co-administration or individual administration of MMR and YF vaccines and documented non-inferiority with co-administration for all antigens compared with individual administration in our ITT population. However, co-administration resulted in significantly lower antibody levels for rubella and YF compared with the values seen with individual administration.

Our results are in agreement with a randomized controlled trial that reported the immune response following individual and co-administration of YF and MR vaccines in children living in The Gambia [15]. In the Gambian study, seroconversion rates for measles, rubella, and YF antigens were non-inferior, using a -10% margin, for children who received co-administered vaccines compared with individually administered vaccines. The authors also noted significantly lower antibody levels for both rubella and YF vaccines but not measles when the vaccines were co-administered. These seroconversion results align with an observational study from France that did not show a statistically significant difference in seropositivity rates between co-administered and sequentially administered YF and measles-containing vaccines [16].

The immunologic findings from The Gambia, France, and our study are different from a randomized controlled trial performed in Brazil where children aged 12–23 months were administered one of two YF vaccines (17DD and 17D-213 sub-strains) at the same time or 28 days following MMR vaccination [5]. In the Brazilian study, both seroconversion rates and antibody levels were significantly lower for mumps, rubella, and YF when the vaccines were co-administered compared with individually administered vaccines. It is not entirely clear why the results differ; however, there were several differences between the studies that could explain some of the discrepancies. First, the follow-up periods to assess immune response after MMR vaccination differed between the co-administration and individual administration groups in Brazil; responses were measured 30 days post-vaccination in the co-administration group and 60 days in the individual group, which allowed more time for antibody development in the individual group [5]. However, this difference in follow-up time did not apply to YF; the immune response was assessed 30 days post-vaccination in all groups. Next, the Brazil study used the 17DD and 17D-213 YF vaccines, while The Gambian study and 98% of children in our study received the 17D-204 YF vaccine. A difference in children’s immune response to YF vaccine strains has been noted by others [17, 18]. On average, the 17DD vaccine has a higher potency than other prequalified vaccines, [19] which might result in greater interference. Another hypothesis is variation in laboratory tests and cut-off points used to classify people as seropositive or seronegative [20]. Finally, it is possible that study populations had different rates of exposure to related viruses (e.g., flaviviruses), and this affected the children’s immune response to the vaccines.

In our study, the decreases in antibody levels for rubella and YF seen when the vaccines were co-administered were not large enough to result in an inferior seroconversion rate in the ITT population—regardless of MMR or YF vaccines being given first or second in the series. However, non-inferiority in YF seroconversion was not demonstrated in the PP analysis, with the lower bound of the 90% CI just crossing the − 5% non-inferiority margin. However, the CI also included zero; therefore, it is also possible that there is no difference among the groups. Overall, this discrepancy is difficult to interpret because the PP analysis was insufficiently powered after the loss of ~ 16% of the ITT participants due to follow-up visits outside of the 28–35 days post-vaccination window.

Interestingly, higher seroconversion rates were seen for measles and rubella when MMR and YF vaccines were co-administered compared to when MMR was administered second in the sequential series. On a related note, seroconversion rates for measles, rubella, and mumps following MMR administration as the second vaccine did not demonstrate non-inferiority when compared to seroconversion rates following MMR administration as the first vaccine. These findings suggest that YF vaccination and its associated immune response notably impact the immune response to MMR vaccine when administered 28–35 days after YF vaccine. These findings could impact considerations for travel vaccinations or vaccination campaigns that target children outside of routine immunization schedules. However, they have limited implications for routine childhood programs if these vaccinations are co-administered or spaced at intervals of two to six months as observed in many routine programs.

While antibody levels were lower for rubella and YF following co-administration compared with individual administration, the geometric mean titers/concentrations achieved for both the co-administration and individual groups were far above the cut-off points used for seropositivity. It is unknown whether the observed magnitude of decrease in antibody levels could affect long-term immunity. Follow-up studies could provide information on whether the co-administration of MMR and YF vaccines has an influence on the long-term dynamics of the respective vaccine-induced IgG antibodies, especially for YF, where only one dose is administered compared to MMR where two doses are administered [1].

The co-administration of vaccines often enables receipt of the vaccines at the earliest recommended age—a strategy that minimizes missed opportunities for vaccination [3]. To avoid any potential interference, MCV and YF vaccines would need to be administered at different vaccination visits, most likely with YF vaccine delayed to a visit after the first recommended MCV dose, because of the current regional measles and rubella elimination goals and transmission dynamics. However, this may result in decreased YF coverage with the delayed vaccine. This decrease was seen in Argentina when YF vaccination was moved from 12 to 18 months in 2014 and led to a drop in YF vaccination coverage from 91% to 2013 to 51% in 2014; [7] while coverage increased each year afterward, it did not catch up to MMR coverage until 2019. Similar decreases in coverage were observed in three other countries in the PAHO region after they moved YF vaccination from 12 months to either 15 or 18 months [7]. In the WHO African Region, all national immunization programs that provide YF vaccine co-administer with M/MR at the 9-month vaccination visit, so there are no data on YF vaccine administration at a later age. However, many countries provide the second dose of M/MR at 15 or 18 months of age, and significant coverage gaps often exist between the first and second dose, even several years after introduction of the second dose [7]. These data underscore that missed opportunities for vaccination should be considered when determining optimal timing for MMR and YF vaccine administration in the childhood vaccination schedule.

One of this study’s strengths was that it was embedded within the national immunization program of Argentina. The results of the ITT analysis demonstrate the vaccinees’ immune response under standard clinic conditions. In addition, with baseline samples on all participants, we were able to exclude the few children that were already seropositive to an antigen before vaccination. However, the study also had several limitations. We encountered challenges completing follow-up visits within the narrow, pre-specified window of 28–35 days post-vaccination; hence our PP population was under-powered for the planned analyses. In addition, we may not have observed peak antibody titers that often occur later after vaccination as seroconversion and antibody titers were measured 4–5 weeks post vaccination. This could impact the magnitude of the differences seen in the antibody concentrations. We did not calibrate the YF antibody titers using an international reference preparation, which makes it difficult to compare our titers to those obtained in Brazil [5] but does allow for general comparison to other studies that also used antibody titers [15, 21–24]. Lastly, our use of PRNT_50_ titers may have caused incorrect classification of the participants with low titers as being seropositive for neutralizing antibodies against the YF virus when in fact the titer was due to cross-reactive antibodies. To help mitigate this, enrollment in the study was temporarily halted during a dengue outbreak in the region.

## Conclusions

This study documented non-inferior seroconversion with co-administration of MMR and YF vaccines for all antigens when compared with individual administration in our ITT population. However, co-administration resulted in significantly lower antibody levels for rubella and YF compared with the values seen with individual administration. The data generated from this study have already contributed to a global policy recommendation. The WHO’s Strategic Advisory Group of Experts on Immunization (SAGE) reviewed the results from this study, along with the studies in Brazil and The Gambia and data on the impact of vaccination schedules on coverage, and decided to retain their recommendation that live, attenuated vaccines can be either co-administered or provided at least four weeks apart from each other [25]. PAHO’s Technical Advisory Group on Vaccine-preventable Diseases subsequently reviewed the same evidence and endorsed the recommendations from SAGE [9]. In line with SAGE and PAHO, we also recommend longer-term follow-up of this and similar cohorts to better understand whether the lower antibody levels observed among studies for rubella and YF following co-administration have any impact on long-term immunity and potential secondary vaccine failure.

## Electronic supplementary material

Below is the link to the electronic supplementary material.

Supplementary Material 1

## Data Availability

De-identified individual participant data that underlie the results reported in this article will be available for sharing along with the study protocol. It will be available to researchers who provide a methodologically sound proposal to achieve the aims in the approved proposal. Data availability will start three months after the publication of this manuscript and will end five years after the publication. Proposals should be directed to jbharris@cdc.gov.
