# Supplementary material for: Immune response to co-administration of measles, mumps, and rubella (MMR), and yellow fever vaccines: a randomized non-inferiority trial among one-year-old children in Argentina
Source: BMC Infect Dis. 2023 Mar 17;23:165. doi: 10.1186/s12879-023-08114-1 (PMC10021967; doi:10.1186/s12879-023-08114-1)
Supplement: Supplementary file 1 — Supplementary Material 1 [file 12879_2023_8114_MOESM1_ESM.docx]

**Additional Material for the manuscript:**

**Immune response to co-administration of measles, mumps, and rubella (MMR), and yellow fever vaccines: a randomized non-inferiority trial among one-year-old children in Argentina**

Table of Contents

[Additional Table 1 2](#_Toc95042237)

[Additional Table 2 4](#_Toc95042238)

[Additional Table 3 5](#_Toc95042239)

[Additional Figure 1 6](#_Toc95042240)

### Additional Table 1

| **Additional Table 1: Comparisons of seroconversion and antibody levels when MMR and YF are co-administered as compared to being administered individually as the 2^nd^ vaccine in a series separated by four weeks** | | | | |
| --- | --- | --- | --- | --- |
|  | **MMR & YF vaccines co-administered*** | **MMR or YF vaccine administered individually as the second vaccine in a series*** | **Difference in seroconversion (co-administration – 2^nd^ vaccine) or**  **p-value for comparison of titers/ concentrations**† | **Interpretation**^3^ |
| ***Measles:***  *Intention-to-treat analysis* | *MMR_1_YF_1_*  *(n=242)* | *YF_1_MMR_2_*  *(n=219)* |  |  |
| Seroconversion | 97.9 (95.3 – 99.1) | 93.2 (89.0 – 95.8) | 4.8 (1.2 – 8.4) | Co-administration has higher seroconversion |
| Antibody concentrations, mIU | 2024 (1705 – 2402) | 1296 (982 – 1710) | 0.018 | Co-administration has higher concentrations |
| *Per protocol analysis* | *(n=203)* | *(n=167)* |  |  |
| Seroconversion | 98.0 (95.0 – 99.2) | 92.8 (87.9 – 95.8) | 5.2 (1.2 – 9.2) | Co-administration has higher seroconversion |
| Antibody concentrations, mIU | 1956 (1629 – 2348) | 1233 (891 – 1708) | 0.068 | Co-administration has higher concentrations |
| ***Rubella***  *Intention-to-treat analysis* | *MMR_1_YF_1_*  *(n=241)* | *YF_1_MMR_2_*  *(n=218)* |  |  |
| Seroconversion | 97.9 (95.2 – 99.1) | 92.7 (88.4 – 95.4) | 5.3 (1.6 – 8.9) | Co-administration has higher seroconversion |
| Antibody concentrations, IU | 35.8 (31.5 – 40.7) | 39.2 (32.9 – 46.7) | 0.013 | Administration as the 2^nd^ vaccine has higher concentrations |
| *Per protocol analysis* | *(n=202)* | *(n=167)* |  |  |
| Seroconversion | 97.5 (94.3 – 98.9) | 93.4 (88.6 – 96.3) | 4.1 (0.1 – 8.1) | Co-administration has higher seroconversion |
| Antibody concentrations, IU | 32.2 (28.0 – 37.1) | 38.5 (31.7 – 46.6) | 0.005 | Administration as the 2^nd^ vaccine has higher concentrations |
| ***Mumps***  *Intention-to-treat analysis* | *MMR_1_YF_1_*  *(n=242)* | *YF_1_MMR_2_*  *(n=217)* |  |  |
| Seroconversion | 96.7 (93.6 – 98.3) | 95.4 (91.7 – 97.5) | 1.3 (-2.1 – 4.7) | Co-administration is non-inferior |
| Antibody concentrations, U | 1807 (1470 – 2220) | 2053 (1584 – 2661) | 0.023 | Administration as the 2^nd^ vaccine has higher concentrations |
| *Per protocol analysis* | *(n=203)* | *(n=167)* |  |  |
| Seroconversion | 96.6 (93.1 – 98.3) | 95.2 (90.8 – 97.6) | 1.3 (-2.4 – 5.1) | Co-administration is non-inferior |
| Antibody concentrations, U | 1746 (1390 – 2192) | 2060 (1529 – 2776) | 0.010 | Administration as the 2^nd^ vaccine has higher concentrations |
| ***Yellow fever*** | *MMR_1_YF_1_* | *MMR_1_YF_2_* |  |  |
| *Intention-to-treat analysis* | *(n=244)* | *(n=214)* |  |  |
| Seroconversion | 96.3 (93.1 – 98.1) | 96.3 (92.8 – 98.1) | 0.05 (-3.2 – 3.3) | Co-administration is non-inferior |
| Antibody titers | 219 (181 – 265) | 278 (229 – 337) | 0.011 | Administration as the 2^nd^ vaccine has higher titers |
| *Per protocol Analysis* | *(n=205)* | *(n=162)* |  |  |
| Seroconversion | 96.1 (92.5 – 98.0) | 96.3 (92.2 – 98.3) | -0.2 (-3.8 – 3.4) | Co-administration is non-inferior |
| Antibody titers | 225 (181 – 279) | 290 (232 – 362) | 0.036 | Administration as the 2^nd^ vaccine has higher titers |
| *Data shown for seroconversion are percentages and 95% CI. Data shown for antibody concentrations are geometric mean concentrations and 95% CI. Data shown for antibody titers are geometric mean titer and 95% CI.  †Data shown are the difference in seroconversion and Farrington-Manning 90% CI or the p-value for Wilcoxon Rank Sum test comparing antibody titers/concentrations. | | | | |

### Additional Table 2

| **Additional Table 2: Comparisons of seroconversion and antibody levels when MMR and YF are administered individually as the 2nd vaccine compared to being administered as the 1st vaccine in a series separated by four weeks** | | | | |
| --- | --- | --- | --- | --- |
|  | **MMR or YF vaccine administered individually as first vaccine in a series*** | **MMR or YF vaccine administered individually as the second vaccine in a series*** | **Difference in seroconversion (2^nd^ vaccine – 1^st^ vaccine) or**  **p-value for comparison of titers/ concentrations†** | **Interpretation^3^** |
| ***Measles*** | *MMR_1_YF_2_* | *MMR_2_YF_1_* |  |  |
| *Intention-to-treat analysis* | *(n=245)* | *(n=219)* |  |  |
| Seroconversion | 96.3 (93.2 – 98.1) | 93.2 (89.0 – 95.8) | -3.2 (-6.7 – 0.4) | Non-inferiority of administration as the 2^nd^ vaccine not shown |
| Antibody concentrations, mIU | 1638 (1323 – 2028 | 1296 (982 – 1710) | 0.409 | No significant difference |
| *Per protocol analysis* | *(n=220)* | *(n=167)* |  |  |
| Seroconversion | 96.4 (93.0 – 98.2) | 92.8 (87.9 – 95.8) | -3.6 (-7.6 – 0.5) | Non-inferiority of administration as the 2^nd^ vaccine not shown |
| Antibody concentrations, mIU | 1569 (1253 – 1964) | 1233 (891 – 1708) | 0.654 | No significant difference |
| ***Rubella*** | *MMR_1_YF_2_* | *MMR_2_YF_1_* |  |  |
| *Intention-to-treat analysis* | *(n=243)* | *(n=218)* |  |  |
| Seroconversion | 94.7 (91.0 – 96.9) | 92.7 (88.4 – 95.4) | -2.0 (-5.9 – 1.9) | Non-inferiority of administration as the 2^nd^ vaccine not shown |
| Antibody concentrations, IU | 40.8 (35.0 – 47.5) | 39.2 (32.9 – 46.7) | 0.873 | No significant difference |
| *Per protocol analysis* | *(n=218)* | *(n=167)* |  |  |
| Seroconversion | 94.5 (90.6 – 96.8) | 93.4 (88.6 – 96.3) | -1.1 (-5.5 – 3.3) | Non-inferiority of administration as the 2^nd^ vaccine not shown |
| Antibody concentrations, IU | 39.4 (33.5 – 46.3) | 38.5 (31.7 – 46.6) | 0.731 | No significant difference |
| ***Mumps*** | *MMR_1_YF_2_* | *MMR_2_YF_1_* |  |  |
| *Intention-to-treat analysis* | *(n=243)* | *(n=217)* |  |  |
| Seroconversion | 97.9 (95.3 – 99.1) | 95.4 (91.7 – 97.5) | -2.6 (-5.6 – 0.5) | Non-inferiority of administration as the 2^nd^ vaccine not shown |
| Antibody concentrations, U | 2252 (1877 – 2701) | 2053 (1584 – 2661) | 0.498 | No significant difference |
| *Per protocol analysis* | *(n=218)* | *(n=167)* |  |  |
| Seroconversion | 98.2 (95.4 – 99.3) | 95.2 (90.8 – 97.6) | -3.0 (-6.4 – 0.5) | Non-inferiority of administration as the 2^nd^ vaccine not shown |
| Antibody concentrations, U | 2319 (1926 – 2792) | 2060 (1529 – 2776) | 0.469 | No significant difference |
| ***Yellow fever*** | *MMR_2_YF_1_* | *MMR_1_YF_2_* |  |  |
| *Intention-to-treat analysis* | *(n=243)* | *(n=214)* |  |  |
| Seroconversion | 97.5 (94.7 – 98.9) | 96.3 (92.8 – 98.1) | -1.3 (-4.4 – 1.9) | Administration as second vaccine is non-inferior |
| Antibody titers | 340 (283 – 408) | 278 (229 – 337) | 0.086 | No significant difference |
| *Per Protocol analysis* | *(n=206)* | *(n=162)* |  |  |
| Seroconversion | 98.1 (95.1 – 99.2) | 96.3 (92.2 – 98.3) | -1.8 (-5.2 – 1.7) | Non-inferiority of administration as the 2^nd^ vaccine not shown |
| Antibody titers | 373 (308 – 452) | 290 (232 – 362) | 0.093 | No significant difference |
| *Data shown for seroconversion are percentages and 95% CI. Data shown for antibody concentrations are geometric mean concentrations and 95% CI. Data shown for antibody titers are geometric mean titer and 95% CI.  †Data shown are the difference in seroconversion and Farrington-Manning 90% CI or the p-value for Wilcoxon Rank Sum test comparing antibody titers/concentrations. | | | | |

| Additional Table 3 **Additional Table 3: Yellow fever seroconversion and post-vaccination antibody titers based on PRNT_90_ test** | | | |
| --- | --- | --- | --- |
|  | **YF and MMR co-administered** | **YF administered as the first vaccine in series** | **YF administered as the second vaccine in series** |
| ***Intention-to-treat analysis*** | *MMR_1_YF_1_ (n=244)* | *YF_1_ MMR_2_ (n=243)* | *MMR_1_YF_2_ (n=215)* |
| Seroconversion | 94.7 (91.1 – 96.9) | 95.9 (92.6 – 97.8) | 96.3 (92.8 – 98.1) |
| Antibody titers | 47.1 (39.9 – 55.7) | 70.7 (60.0 – 83.4) | 63.1 (53.7 – 74.2) |
| ***Per protocol analysis*** | *(n=205)* | *(n=206)* | *(n=163)* |
| Seroconversion | 94.2 (90.1 – 96.6) | 96.6 (93.2 – 98.3) | 96.3 (92.2 – 98.3) |
| Antibody titers | 48.2 (39.9 – 58.3) | 76.8 (64.5 – 91.4) | 67.5 (56.1 – 81.3) |
| Seroconversion data are % (95% Confidence Interval).  Antibody titers are geometric mean titer (95% CI). | | | |

Text for Supplementary Table 3: The neutralizing titers below were assessed using the plaque reduction neutralization test with a cut-off of 90% (PRNT_90_)^[[1]](#footnote-2)^. Compared to the PRNT_50_ used in the main analyses, a 90% cut-off decreases the sensitivity but improves the specificity of the test by decreasing the likelihood that low level titers caused by cross-reactive antibodies to related viruses will be detected. While PRNT_90_ is often used for diagnostic purposes, PRNT_50_ is more often used in studies with pre and post vaccination samples as it provides a more accurate result from the linear proportion of the titration curve^[[2]](#footnote-3)^.

### Additional Figure 1


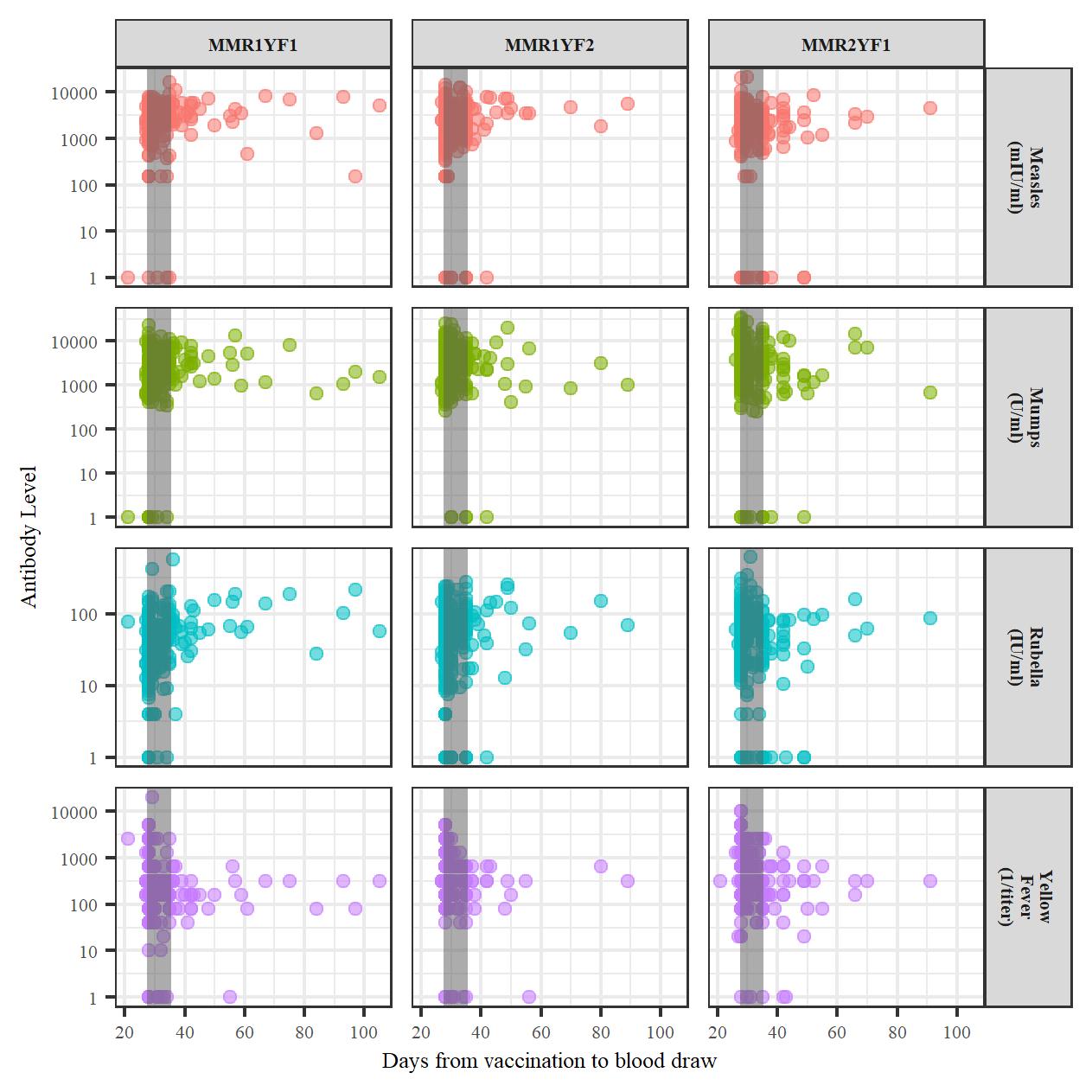


**Additional Figure 1:** Antibody concentrations (against measles, rubella, mumps) or antibody titer (against yellow fever) over time from vaccination to blood sample collection. The window for inclusion in the per-protocol analysis is shown in gray. Overall, antibody concentrations/titers remained fairly constant as time from vaccination to specimen collection increased.

1. Beaty BJ, Calisher CH, Shope RE. Arboviruses. In: Lennette EH, Lennette DA, Lennette ET, eds. Diagnostic Procedures for Viral, Rickettsial, and Chlamydial Infections. Washington, D.C.: American Public Health Association; 1995. [↑](#footnote-ref-2)
2. WHO 2007 Guidelines for plaque reduction neutralization testing of human antibodies to dengue virus. Available at: <https://apps.who.int/iris/bitstream/handle/10665/69687/who_ivb_07.07_eng.pdf?sequence=1&isAllowed=y>. Accessed on 9 March 2020. [↑](#footnote-ref-3)
